# Propofol TCI or sevoflurane anesthesia without muscle relaxant for thoracoscopic thymectomy in myasthenia gravis patients: a prospective, observational study

**DOI:** 10.1186/s12871-023-02296-6

**Published:** 2023-10-21

**Authors:** Vo Van Hien, Nguyen Huu Tu, Nguyen Dang Thu

**Affiliations:** 1 Department of Anesthesiology, Military Hospital 103, Vietnam Military Medical University, No.261 Phung Hung Street, Ha Dong District, Hanoi, 12108 Vietnam; 2 Department of Anesthesiology, National Burn Hospital, Vietnam Military Medical University, No.263 Phung Hung Street, Ha Dong District, Hanoi, 12108 Vietnam; 3 Department of Anesthesia and Critical Care, Hanoi Medical University, No.1 Ton That Tung Street, Dong Da District, Hanoi, 116177 Vietnam; 4 Department of Musculoskeletal Functional Research and Regeneration, Graduate School of Biomedical and Health Sciences, Hiroshima University, 1-2-3 Kasumi, Minami-Ku, Hiroshima City, Hiroshima, 734-8553 Japan

**Keywords:** Myasthenia gravis, Thoracoscopic thymectomy, Propofol TCI, Sevoflurane, Without muscle relaxant

## Abstract

**Background:**

Myasthenia gravis (MG) patients interact unpredictably with anesthetic agents, including neuromuscular blocking agents. Here, we investigate the effectiveness of general anesthesia without muscle relaxants using either propofol via target-controlled infusion systems (TCI) or sevoflurane in MG patients undergoing thoracoscopic thymectomy.

**Methods:**

This prospective, open-label, observational study was conducted in a university hospital. We included 90 myasthenic patients undergoing thoracoscopic thymectomy with general anesthesia. Patients received induction and maintenance anesthesia with propofol TCI (group P, *n* = 45) or induction with propofol 2–3 mg.kg^−1^ and maintenance anesthesia with sevoflurane (group S, *n* = 45). In both groups, the procedure was performed under the guidance of entropy with sufentanil but not a muscle relaxant. Intubation conditions, hemodynamic changes, respiratory function, neuromuscular transmission, arterial blood gas, and complications were evaluated.

**Results:**

All patients achieved good intubation conditions. Hemodynamic instability was more frequent in group S than in group P, mostly in the induction stage, and was controllable. The reduction in the intraoperative train-of-four ratio from baseline at 30 min, 60 min, and 90 min in group S was 10.3%, 14.2%, and 14.3%, respectively, significantly higher than that in group P (6.8%, 7.2%, and 8.4%, respectively), which completely recovered at the end of the surgery. All patients were extubated in the operating room without complications. No other significant differences between the groups were observed.

**Conclusions:**

Anesthesia with propofol TCI or sevoflurane without muscle relaxants in MG patients offered safe and effective conditions for thoracoscopic thymectomy. Sevoflurane achieved higher levels of intraoperative muscular relaxation than propofol TCI. Postoperative neuromuscular function was not affected by these anesthetics.

## Introduction

Myasthenia gravis (MG) is an autoimmune disorder induced by neurotransmission defects at the neuromuscular junction characterized by muscle weakness and fatigue [1]. Along with immunotherapies, thymectomy has been recommended as the standard treatment therapy for MG patients [2, 3].

General anesthesia in myasthenic patients requires careful perioperative management due to its unpredictable interaction with anesthetic agents, particularly nondepolarizing neuromuscular blocking agents (NMBAs) [4]. Residual paralysis risks precipitating a postoperative myasthenic crisis or respiratory failure, while excess reversal with anticholinesterases may lead to a cholinergic crisis [5]. Although sugammadex is a rapid reversal of rocuronium- or vecuronium-induced neuromuscular block [6–10], unsuccessful cases using sugammadex to reverse rocuronium in patients with MG were reported [11, 12], and sugammadex was not always available in some circumstances. Therefore, anesthesia without muscle relaxation for surgery in MG patients have still been considered [13].

Sevoflurane is a potent inhaled agent with low blood solubility and less airway excitation. It is commonly used for anesthesia due to its easily adjustable effect concentration and rapidly eliminated at the end of surgery [14]. Meanwhile, propofol is a short-acting anesthetic that can be infused continuously or via target-controlled infusion (TCI) systems. The latter technique provides the estimated effective concentration of the drug in the brain or in plasma and automatically adjusts the infusion rate to achieve the desired concentration [15]. Both anesthetics could be advantageous for patients with MG.

Here, we investigate the effectiveness of propofol TCI and sevoflurane for general anesthesia maintenance without muscle relaxants in MG patients undergoing thoracoscopic thymectomy and document perioperative complications.

## Subjects and methods

### Study design and patients

This single-center, open-label, observational study was approved by Hanoi Medical University’s ethics committee (No.168-QĐ/ĐHYHN) and Military Hospital 103. Written informed consent was obtained from all patients and this study was reported in accordance with the STROBE statement. Between November 2018 and April 2021, we included 90 myasthenic patients undergoing thoracoscopic thymectomy with general anesthesia. Patients were performed induction and maintenance anesthesia with propofol TCI (group P, *n* = 45) or induction with propofol 2–3 mg.kg^−1^ and maintenance anesthesia with sevoflurane (group S, *n* = 45). MG patients were definitively diagnosed using anticholinesterase and electroneuromyographic tests and clinically classified according to the Myasthenia Gravis Foundation of America (MGFA) [16]. Current anticholinesterase and steroid therapy were stopped on the morning of surgery and restarted the next day. Chest computed tomography scan, spirometry, arterial blood gas analysis, and other routine preoperative examinations were performed.

### Anesthesia procedure

Intraoperative monitoring included electrocardiogram, invasive arterial blood pressure, pulse oximetry, expiratory gas analysis (end-tidal CO_2_-EtCO_2_; end-tidal sevoflurane-EtSevoflurane), and entropy including response entropy (RE) and state entropy (SE) index (Datex Omeda S/5 Advance, GE, USA). Neuromuscular monitoring was recorded from the adductor pollicis muscle using TOF (train-of-four)-WatchsSX (Organons, Oss, the Netherlands) with stimulation of the ulnar nerve of the left warmed and immobilized forearm. Soon after losing consciousness with anesthetic induction, we calibrated the baseline twitch amplitude. Then, supramaximal stimuli (60 mA for 200 µsec) at 20-s intervals were performed, and the acceleration of the thumb was measured. During the train, each twitch response value and TOF ratio (T4/T1) were recorded.

All patients were oxygenated with 100% oxygen for five minutes before induction but were not premedicated. Anesthesia was induced with sufentanil at 0.5 µg.kg^−1^ and propofol via the TCI system at Ce 5–7 µg.ml^−1^ (target effect concentration; Schneider model; group P) or the manual injection method at 2–3 mg.kg^−1^ (group S). Lidocaine 2% and 10% spray were used for topical anesthesia of the pharynx and larynx. When patients lost the eyelash reflex and the SE value was 50 or lower, a Univent endotracheal tube (Fuji Systems, Japan) was placed and checked with a flexible bronchoscope. The time to achieve these events, duration, and condition of intubation (according to Thomas Fuchs-Buder et al. [17]) were documented.

The ventilator was initially set in A/C mode (FiO_2_ = 60%, respiratory rate of 14 breaths.min^−1^, tidal volume of 8–10 ml.kg^−1^, inspiration-expiration ratio of 1:2) during the two-lung ventilation period and adjusted during the one-lung ventilation period to maintain the EtCO_2_ between 30–35 mmHg, SpO_2_ > 95% and P_peak_ < 30 cmH_2_O. All patients received lactated Ringer's solution (8–10 ml.kg^−1^.hr^−1^) during the procedure.

The maintenance of anesthesia was performed under the guidance of entropy. The Ce (group P) and EtSevoflurane (group S) were adjusted to maintain the SE values in a range of 40–60 during the operation procedure and then withdrawn immediately after the final skin closure. In both groups, sufentanil was infused continuously at 0.2 µg.kg^−1^.hr^−1^, supplemented with a dose of 0.1 µg.kg^−1^ as needed, and infusion was stopped 20 min before completing the surgery. The nasopharyngeal temperature was maintained at 36.5 °C –37 °C with a heating blanket. The patient was positioned in a 45° lateral decubitus orientation, either on the left or right side, depending on the anatomical characteristics of the thymoma. Selective one-lung ventilation was established by inflating the endobronchial blocker balloon before making any skin incisions. Next, three trocars were introduced, and the tumor was carefully dissected and removed. A drainage tube was inserted before re-expanding the collapsed lung, and the incision was subsequently closed. At the end of the surgery, patients were extubated in the operating room unless they did not meet the extubation criteria. Postoperative analgesia was managed with paracetamol (15 mg.kg^−1^) and nefopam (20 mg).

Hemodynamic changes (heart rate-HR, mean arterial pressure-MAP), dose of anesthetic agents, and entropy value (RE, SE) were recorded prior to anesthesia (t0-baseline), at loss of consciousness (t1), before intubation (t2), right after intubation (t3), 5 min after intubation (t4), right before skin incision (t5), right after skin incision (t6), 5 min after skin incision (t7), inserting a trocar into the pleural space (t8), thymus dissection (t9), inserting drainage tube into pleural space (t10), squeezing the Ambu Bag to inflate the lungs (t11), before skin closure (t12), finishing surgery (t13), before extubation (t14), 5 min after extubation (t15), and 30 min after extubation (t16). TOF ratios were recorded at induction (baseline), 30 min, 60 min, 90 min thereafter, and immediately before extubation. Anesthesia and surgery time, time from stopping anesthetic agents to eye-opening, extubation, and full recovery of awareness were also documented.

Bradycardia (HR < 50 beats/min) and/or hypotension (MAP < 80% of the baseline value) were treated with atropine and/or ephedrine. Arterial blood gas was checked at both two-lung and one-lung ventilation times during the intraoperative period, 2 h after extubation, and thrice for a period of 3 days postoperatively. Pulmonary function was also assessed with spirometry during the 3 days after the operation. Perioperative complications were recorded.

### Statistical analysis

Data are presented as the mean with range, standard deviation (SD), or 95% confidence interval (95% CI). Statistical analyses were performed using the software package SPSS (version 19.0, IBM Japan, Tokyo, Japan). Shapiro‒Wilk and Levene’s tests were used to assess the normality and homogeneity of variance, respectively. Significant differences between groups were evaluated using Student’s t test, Mann‒Whitney U test, and two-way ANOVA with Bonferroni post hoc test. Chi-squared or Fisher’s exact tests were used for categorical variables. Differences were considered statistically significant at *p* < 0.05.

## Results

No significant differences were noted among the groups regarding the characteristics of patients (Table 1). The time to achieve intubation criteria was shorter in group P than in group S (Table 2). The most frequent grades of the Cormack and Lehane scale recorded during laryngoscopy were I and II. Intubation conditions were excellent in 41 of the 45 patients in group P and in 43 of the 45 patients in group S with a single attempt, and the remaining patients were successfully intubated on the second attempt after supplementing propofol. The duration of intubation, anesthesia, or surgery was similar in both groups (Table 2).

**Table 1:** Characteristics of patients

	**Group P** **(*n* = 45)**	**Group S** **(*n* = 45)**	***p* value**
Gender (male/female)	20/25	21/24	0.832^α^
Age – years (mean ± SD)	43.2 ± 15.1	40.7 ± 11.9	0.862^β^
Body weight – kg (mean ± SD)	56.7 ± 7.9	53.3 ± 9.4	0.440^β^
Height – m (mean ± SD)	161.6 ± 6.9	161.4 ± 6.8	0.951^β^
MGFA classification – no. (%)			
I	15 (33)	13 (28)	0.109^α^
II_a_	27 (60)	22 (49)	
II_b_	3 (7)	10 (22)	
Disease duration-months (mean, range)	34.3 (1–96)	28.8 (1–108)	0.549^γ^
Thyroma (yes/no)	41/4	37/8	0.353^δ^
Pre-operative treatment			
Pyridostigmine – no. dosage-mg.day^−1^ (mean, range)	45 263 (60–600)	45 224 (60–540)	0.226^β^
Prednisone – no	5	10	0.157^α^
Plasmapheresis – no	6	7	0.764^α^

*MGFA* Myasthenia Gravis Foundation of America

^α^Chi-squared test

^β^Student’s t test

^γ^Mann‒Whitney U test

^δ^Fisher’s exact test

**Table 2:** Perioperative events

	**Group P** **(*n* = 45)**	**Group S** **(*n* = 45)**	***p ***value
Time to lose eyelid reflex (s)	96 (45–120)	117 (57–155)	< 0.001^β^
Time to achieve SE < 50 (s)	129 (90–170)	147 (100–186)	< 0.001^β^
Intubation duration (min)	6.3 (4–10)	6.6 (4–12)	0.332^β^
Surgery duration (min)	116 (60–180)	119 (60–145)	0.309^β^
Anesthesia duration (min)	148 (90–210)	155 (90–180)	0.112^β^
Time to eye-opening (min)	10 (7–15)	12 (8–15)	< 0.001^β^
Time to extubation (min)	13.4 (9.5–18)	12.7 (8–17)	0.223^β^
Self-awareness time (min)	15 (11–19)	14 (9.5–18)	0.016^β^
Bradycardia – no. (%)	10 (22)	25 (56)	0.001^α^
Hypotension – no. (%)	11 (24)	21 (47)	0.028^α^
Intraoperative unexpected movement – no. (%)	5 (11)	1 (2)	0.203^δ^

Data regarding time represented as the mean (range)

^β^Student’s t test

^α^Chi-squared test

^δ^Fisher’s exact test

**Fig. 1:**
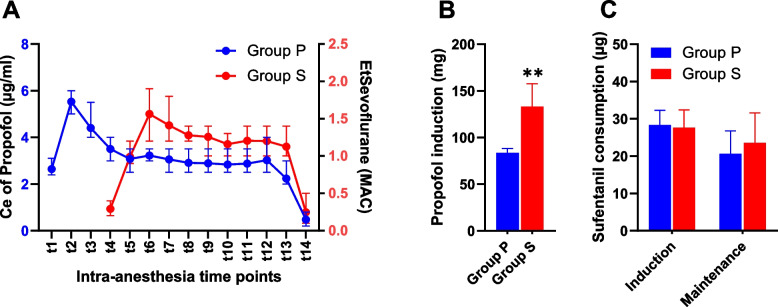
Intraoperative anesthetic agents. **A** Concentration effect (Ce) of propofol during the induction (t1–t3) and maintenance stages (t4–t14) in group P and age-adjusted minimum alveolar concentration (MAC) of sevoflurane in the maintenance stage in group S; data are represented as the mean with range. **B** Propofol used for induction and (**C**) sufentanil consumption in each period of anesthesia; data are represented as the mean with SD. **p < 0.01, Student’s t test

**Fig. 2:**
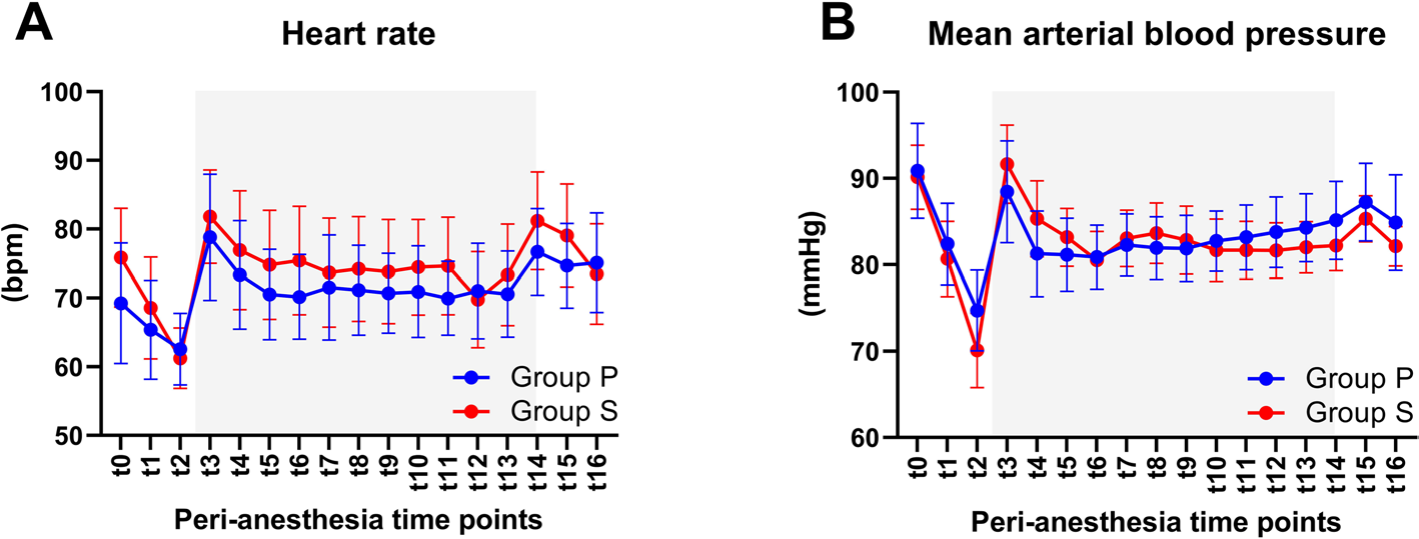
Peri-anesthesia hemodynamic changes. **A** Heart rate and (**B**) mean arterial blood pressure change during the preanesthesia (t0), induction (t1–t2), maintenance (t3–t13), and emergence (t14–t16) stages of anesthesia. Data are represented as the mean with SD. Gray boxes show the maintenance stage of anesthesia

Propofol use for induction was 83.7 ± 4.6 mg in group P and 133.1 ± 24.5 mg in group S, with a *p* value < 0.001 (Fig. 1B). The Ce of propofol in group P and the EtSevoflurane in group S used for maintaining anesthesia was in the range of 2.5–4 µg.ml^−1^ and 1–2 age-adjusted MAC, respectively (Fig. 1A). The sufentanil consumption was comparable among groups (Fig. 1C). The heart rate and arterial blood pressure significantly decreased during the induction period but returned to the normal range after intubation with minimal changes throughout the entire procedure (Fig. 2); bradycardia and hypotension were more frequent in group S than in group P (Table 2). This hemodynamic instability was controllable with atropine and/or ephedrine.

**Fig. 3:**
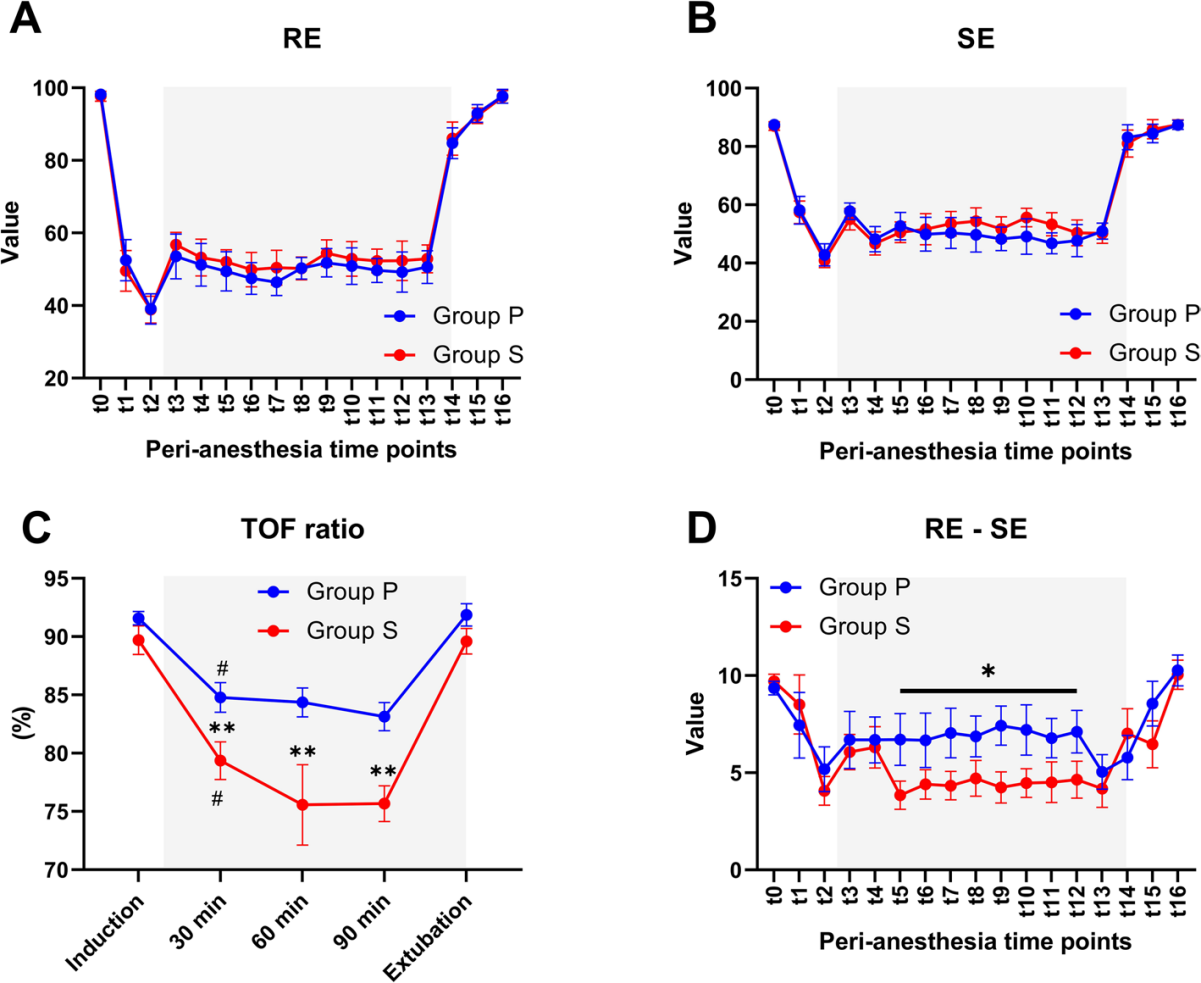
The depth of anesthesia and neuromuscular blockade. Response entropy (RE; **A**), state entropy (SE; **B**), and the difference in RE and SE values (RE-SE; **D**) during the preanesthesia (t0), induction (t1–t3), maintenance (t4–t13), and emergence (t15–t16) stages of anesthesia. **C** Train-of-four (TOF) value in the maintenance stage. Data are represented as the mean with 95% CI. The differences were estimated by two-way repeated-measures ANOVA with Bonferroni post hoc test (*p < 0.05; **p < 0.01; between groups, #*p* < 0.05; maintenance vs. induction stage). Gray boxes show the maintenance stage of anesthesia

The same depth of anesthesia level (Fig. 3A, B) and the same baseline neuromuscular transmission were observed. Compared to the baseline value, the TOF ratios in both groups were significantly decreased after induction of anesthesia with a *p* value < 0.05. The TOF ratio in group P decreased by 6.8%, 7.2%, and 8.4% at 30 min, 60 min, and 90 min, respectively. Sevoflurane provided a higher level of muscle relaxants, with values in group S of 10.3%, 14.2%, and 14.3% (*p* < 0.01, compared between groups; Fig. 3C). A lower value in the difference between RE and SE was observed in group S than in group P (*p* < 0.05 at the maintenance of anesthesia time points; Fig. 3D). All the above parameters in both groups returned to baseline values at the end of surgery. Intraoperative unexpected movement was gentle and not effective for the surgical procedure (Table 2).

**Fig. 4:**
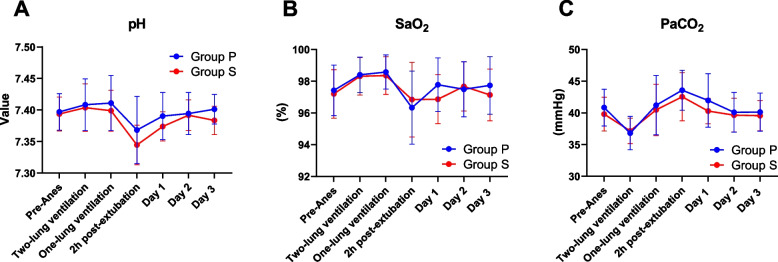
Perioperative arterial blood gas results. The pH value (**A**), oxygen saturation (SaO_2_, **B**), and partial pressure of carbon dioxide (PaCO_2_, **C**) at preanesthesia (preanesthesia), intraanesthesia, and three days postanesthesia were comparable between groups. Data are represented as the mean with 95% CI

**Fig. 5:**
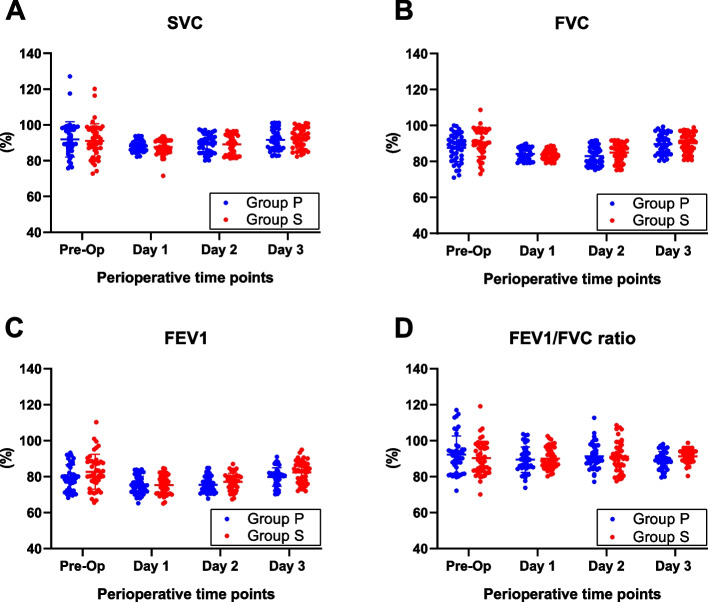
Perioperative pulmonary function spirometry results. The slow vital capacity (SVC; **A**), forced vital capacity (FVC; **B**), forced expiratory volume in the first second (FEV1; **C**), and FEV1/FVC (**D**) values preoperatively (preop) and three days postanesthesia were comparable between groups. Dots represent individual values

After finishing the surgery, the time to eye opening and self-awareness were statistically difference between group P and group S; however, these differences were not clinically significant, and the time to extubation was similar in both groups (Table 2). All patients were extubated in the operating room. The arterial blood gas (pH value, SaO_2_, and PaCO_2_; Fig. 4) and spirometry (slow vital capacity-SVC, forced vital capacity-FVC, forced expiratory volume in the first second-FEV1, and FEV1/FVC ratio; Fig. 5) results showed minimal change between each time point check but no significant difference between groups. No patients suffered from respiratory failure, myasthenia, cholinergic crisis or other major cardiovascular complications.

## Discussion

Our main findings indicate that both propofol TCI and sevoflurane provide effective and safe anesthesia for thoracoscopic thymectomy in MG patients without muscle relaxants. Maintaining anesthesia with sevoflurane achieved higher levels of muscular relaxation than with propofol TCI.

In recent years, the wide application of thoracoscopic and advanced anesthetic techniques for myasthenic patients has a good track record for safety [3]. Less invasive thymectomy approaches result in less intraoperative tissue damage, less intraoperative blood loss, a decreased inflammatory cytokine response, less anesthetic drug consumption, and less postoperative pain [18]. Thus facilitating a faster recovery of these patients. The advanced anesthetic delivery method and short-acting agents also help general anesthesia promote early recovery and minimize respiratory depression [19]. The anesthetic management of MG is individualized, taking into consideration the disease; its treatment; and the effects of surgery, anesthesia, and associated medications.

Induction with propofol and opioids can provide satisfactory conditions for tracheal intubation in MG without muscle relaxants [20]. In the present study, the intubation condition was good in all patients with propofol both via TCI and manual injection. Propofol displays rapid onset, short duration of action, and suppressed airway reflexes. After inducing anesthesia with propofol and sufentanil, patients quickly achieved the appropriate anesthesia level. Although the TOF ratio was approximately 90% at the time of intubation, most patients were successfully intubated after a single attempt. Using lidocaine spray to anesthetize the upper airway, the coughing reflex was also minimized [21]. Although hemodynamic changes were observed in both groups, the TCI technique seems to show less variable heart rate and blood pressure than the manual injection method. This can be explained by the difference in the dosage and infusion rate of propofol in the two methods. The adverse hemodynamic events were successfully treated with atropine and/or ephedrine. Total intravenous anesthesia for the management of myasthenics has also been reported with high freaquency hemodynamic instability in older patients [22].

We compared two techniques for maintaining anesthesia with propofol TCI and sevoflurane. These are the two most commonly used anesthetics with safe properties, ensuring deep anesthesia, fewer hemodynamic changes, and good quality of recovery. In this study, we used entropy to monitor the depth of anesthesia, and the drug concentrations were adjusted to achieve an appropriate anesthesia level [23, 24]. Meanwhile, propofol infused with computer-assisted TCI systems provides an optimal dose of anesthetic for individual patients, consequently maintaining a stable level of anesthesia. Sevoflurane was adjusted following the end-tidal concentration parameter. In terms of hypnosis, both techniques provide satisfactory anesthesia conditions with minimal hemodynamic changes and rapid recovery of consciousness. However, the results of this study showed that MG patients are more sensitive to sevoflurane than to propofol. In the maintenance of anesthesia, the TOF values of the sevoflurane group were significantly lower than those in the induction period and lower than the corresponding value of the propofol TCI group. The difference in muscular relaxation level was not only displayed in the TOF index but also in the difference between RE and SE values during maintenance. SE is derived from the isoelectric electroencephalogram (EEG) signal representing brain activity, while RE is calculated based on the analysis of the power spectrum of the EEG signal and the interaction between different frequency components, including frontal electromyography [25]. Therefore, a lower value in the difference between RE and SE might present a higher level of facial muscular relaxation. A fade of the TOF ratio in myasthenic patients who underwent anesthesia with sevoflurane can also facilitate the surgeon’s work and guarantee the immobility of the patients during the operative procedure. Della Rocca G et al. investigated the perioperative conditions of general anesthesia without muscle relaxants, using either propofol or sevoflurane, for thymectomy in MG patients [21]. Although propofol was administered as a continuous infusion without monitoring the depth of anesthesia, they found that both techniques provided good intraoperative conditions and allowed for early extubation in the operating room after a trans-sternal procedure. In the present study, similar anesthetic agents were utilized in patients with comparable characteristics. However, these patients underwent laparoscopic thoracic surgery, which necessitated one-lung ventilation during the surgical procedure and an aggressive intraoperative movement may interrupt the procedure or even cause severe surgical complications. In addition, propofol was administered using the TCI method, and anesthesia maintenance was guided by state entropy parameters in all patients. Evidence from both studies suggests that anesthesia without muscle relaxants with propofol or sevoflurane is feasible for thymectomy in MG patients either undergoing trans-sternal or laparoscopic approaches.High concentrations of volatile anesthesia affect neuromuscular transmission, and these agents have been reported to provide dose-dependent neuromuscular relaxation in patients with MG. In our study, a significant decrease in T4/T1 at approximately 1–1.5 MAC sevoflurane was observed. Nitahara K et al. [26] found that the degree of reduction in the TOF index depends on the concentration; the baseline index of 81% was reduced to 64% at 1.7% sevoflurane and to 43% at 3.4% sevoflurane. However, 1.9 MAC of halothane and isoflurane had no inhibitory effects on T4/T1 in control patients [27]. Desflurane at 1–1.5 MAC resulted in a significant decrease in the TOF ratio compared with propofol in MG patients [28] and at 1.67 MAC in volunteers [29]. The potential mechanism of sevoflurane at high concentrations affects neuromuscular transmission by prolonging the refractory period and inhibiting synaptic acetylcholine mobilization and sensitization of the receptor [30]. The site of action is proposed to be both pre- and postsynaptic [31]. However, the cause of the gradual loss of muscle contraction responses after continuous stimulation (TOF) is due to an inhibitory effect on acetylcholine release at the presynaptic membrane. Therefore, sevoflurane was suggested to have a significant depressant action at presynaptic neuromuscular junctions.

In the emergence from anesthesia period, TOF ratios returned to the initial value, and there was no difference between the groups. The rapid elimination of propofol and the rapid kinetics (low blood-gas solubility coefficient) of sevoflurane allow rapid recovery of consciousness and early extubation [21]. In fact, extubation was performed in the operating room approximately 13 min after discontinuing anesthetic agents, without significant postoperative complications. Neither volatile nor intravenous anesthetics cause prolonged muscle relaxation in MG patients. The recovery time was similar in both groups, and the quality of recovery was good, as shown by a short time of self-awareness. Patients in both groups displayed adequate return of spontaneous breathing, airway reflexes, and muscle strength. Satisfactory postoperative pain relief facilitates respiratory function and prevents postoperative complications related to MG, such as myasthenic crisis or cholinergic crisis [32]. Our results reveal a stable pH value and PaCO_2_ during the 3-day postoperative period; no hypoxemia or respiratory failure cases were observed at that time. Chevalley C et al. [33] found that postoperative artificial ventilation incidence is four times higher in patients with MG than in those without MG when giving balanced anesthesia, and that figure was fourteen times when using muscle relaxants. The advantages of early recovery might increase the treatment effectiveness of MG and reduce the length of hospital stay [34].

The main limitations of the present study are that it was conducted at a single center and included patients with MGFA classification I or II with initial symptoms of the disease. There is no evidence to draw conclusions about the effect of anesthesia with propofol or sevoflurane on MG patients with MGFA classification III or higher. We knew that the need for postoperative ventilation is dependent on the operation and the preoperative clinical extent of the MG with risk factors including disease duration longer than 6 years, comorbid respiratory diseases, a low vital capacity (< 50%) [35], and transsternal thymectomy [36]. Hence, from an anesthesia aspect, avoiding neuromuscular blocking drugs when possible, using short-acting anesthetics under guidance of anesthesia level monitoring and adequate postoperative pain control are suggested to improve the clinical outcomes of MG patients with surgical treatment.

The results of this study might suggest a general anesthetic technique using propofol via TCI system for induction and sevoflurane for maintenance without muscle relaxants for MG patients undergoing other surgery rather than thoracoscopic thymectomy.

## Conclusion

In conclusion, we can confirm that anesthesia with propofol TCI or sevoflurane without muscle relaxants in myasthenic patients offered good intra- and postoperative conditions for thoracoscopic thymectomy. Sevoflurane achieved higher levels of muscle relaxation than propofol TCI for the maintenance of anesthesia. Postoperative neuromuscular function was not affected by these anesthetics.

## Data Availability

The data supporting this study’s findings are available from the corresponding author upon reasonable request.

## Abbreviations

MG: Myasthenia gravis
TCI: Target-controlled infusion
NMBAs: Neuromuscular blocking agents
MGFA: Myasthenia Gravis Foundation of America
EtCO_2_: End-tidal carbon dioxide
EtSevoflurane: End-tidal sevoflurane
RE: Response entropy
SE: State entropy
TOF: Train-of-four
A/C: Assist/Control
FiO2: Fraction of inspiratory oxygen
SpO_2_: Saturation of peripheral oxygen
HR: Heart rate
MAP: Mean arterial pressure
MAC: Minimum alveolar concentration
PaCO_2_: Arterial partial pressure of carbon dioxide
SVC: Slow vital capacity
FVC: Forced vital capacity
FEV1: Forced expiratory volume in the first second
EEG: Electroencephalogram
